# Targeting IGF2BP2 Promotes Differentiation of Radioiodine Refractory Papillary Thyroid Cancer via Destabilizing RUNX2 mRNA

**DOI:** 10.3390/cancers14051268

**Published:** 2022-03-01

**Authors:** Ri Sa, Rui Liang, Xian Qiu, Ziyan He, Zhiyan Liu, Libo Chen

**Affiliations:** 1 Department of Nuclear Medicine, Shanghai Jiao Tong University Affiliated Sixth People’s Hospital, 600# Yishan Road, Shanghai 200233, China; sarihoo012@sjtu.edu.cn (R.S.); 120725910638@sjtu.edu.cn (X.Q.); he-ziyan@sjtu.edu.cn (Z.H.); 2 Department of Nuclear Medicine, The First Hospital of Jilin University, 1# Xinmin Street, Changchun 130021, China; 3 Department of Geriatrics and National Clinical Research Center for Geriatrics, West China Hospital, Sichuan University, 37# Guo Xue Xiang, Chengdu 610041, China; liangrui67@wchscu.cn; 4 Department of Pathology, Shanghai Jiao Tong University Affiliated Sixth People’s Hospital, 600# Yishan Road, Shanghai 200233, China

**Keywords:** papillary thyroid cancer, differentiation, IGF2BP2, RUNX2, sodium/iodide symporter

## Abstract

**Simple Summary:**

Differentiation therapy is one of the most promising treatment approaches for radioiodine refractory papillary thyroid cancer (RR-PTC). In this study, we found that insulin-like growth factor 2 mRNA-binding protein 2 promoted dedifferentiation of PTC via integrating to 3′-untranslated regions of runt-related transcription factor 2, which bound to the promoter region of sodium/iodide symporter, downregulating its expression.

**Abstract:**

N6-methyladenosine (m6A) regulators play an important role in multiple biological and pathological processes of radioiodine refractory papillary thyroid cancer (RR-PTC). However, the function of m6A regulators in differentiation of RR-PTC remains unclear. In this study, online data, clinical samples, and RR-PTC cell lines (K1 and TPC1) were used to identify the m6A regulators that contributed to the differentiation of RR-PTC. Insulin-like growth factor 2 mRNA-binding protein 2 (IGF2BP2) was found to be associated with thyroid-specific genes in online data analyses, and metastatic PTCs with high expression of IGF2BP2 were prone to be ^131^I-nonavid in clinical analyses. Furthermore, targeting IGF2BP2 increased ^125^I uptake in RR-PTC cell lines and enhanced the sodium/iodide symporter (NIS) expression. Mechanistically, IGF2BP2 bound to the m6A modification site of runt-related transcription factor 2 (RUNX2) 3′-UTR and enhanced the RUNX2 mRNA stability. Moreover, RUNX2 could bind to the promoter region of NIS to block the differentiation of RR-PTC. Together, these results demonstrated that IGF2BP2 represents a diagnostic marker for RR-PTC, suggesting a novel differentiation therapeutic strategy of targeting IGF2BP2.

## 1. Introduction

Thyroid cancer is the most common endocrine malignancy, and papillary thyroid carcinoma (PTC) represents the most frequent pathological type, at nearly 85% of cases [1]. Radioiodine (^131^I) therapy is the first line treatment regimen for local recurrence and/or distant metastatic ^131^I-avid disease, keeping the tumors in the state of “tumor dormancy” for a much longer time, even forever, to avoid disease progression with few adverse effects. Nevertheless, approximately 60% foci are ultimately named as radioiodine refractory papillary thyroid carcinoma (RR-PTC) owing to the loss of iodide uptake and organification, which is controlled by thyroid-specific iodide-handling genes, including sodium/iodide symporter (NIS), thyroid peroxidase (TPO), thyroid-stimulating hormone receptor (TSHR), and thyroglobulin (TG) [2]. Dedifferentiation alters the mRNA or protein levels of iodide-handling genes and renders the tumor refractory to ^131^I therapy. Dismal prognosis has made RR-PTC a clinical challenge and research focus.

Differentiation therapy has been deemed a promising strategy which facilitates RR-PTC to recover the ability of ^131^I uptake and leads to prolonged survival times for RR-PTC patients. To date, several treatment strategies have been explored for the differentiation therapy of RR-PTC. For example, several tyrosine kinase inhibitors such as Selumetinib, Dabrafenib, and Vemurafenib, etc., have been adopted for the differentiation/redifferentiation therapy of RR-PTC [3], since genetic and epigenetic alterations in the mitogen-activated protein kinase (MAPK) and phosphatidylinositol 3-kinase (PI3K)/protein kinase B (AKT)/mammalian (or mechanistic) target of rapamycin (mTOR) pathways are well accepted as the main contributors to ^131^I refractoriness [4]. Unfortunately, accumulating evidence has shown that their clinical effectiveness remains insufficient [5]. Hence, the discovery of new key mechanisms involving in the dedifferentiation of PTC are mandatory, which could assist the RR-PTC patients regain benefit from ^131^I treatment.

RNA modification plays a critical role in cell differentiation [6,7]. N6-methyladenosin (m6A) is one of the most pervasive modifications of mRNA in eukaryotes, taking control of many biological processes. This modification is regulated by the m6A methyltransferases (writers) methyltransferase-like 3 (METTL3), METTL14, METTL16, Zinc finger CCCH domain-containing protein 13 (ZC3H13), and Homo sapiens RNA binding motif protein 15 (RBM15); demethylases (erasers) fat mass- and obesity-associated protein (FTO) and alkB homolog 5 (ALKBH5); and binding proteins (readers) YTH domain family (YTHDF) proteins such as YTHDF1-3 and YTHDC1-2, and insulin-like growth factor 2 messenger RNA-binding proteins (IGF2BP1-3) [8]. These proteins are frequently upregulated or downregulated in human cancer tissues to control cell differentiation via altering splicing, RNA processing, protein translation, microRNA binding, and RNA-protein interaction [9]. For example, METTL14-dependent m6A methylation-mediated hepatocyte nuclear factor 3γ mRNA reduction rendered hepatocellular carcinoma dedifferentiation [10]. In another study, FTO inhibited m6A levels of Adper Single Bond 2 and retinoic acid receptor alpha and inhibited all-trans-retinoic acid-induced acute myeloid leukemia cell differentiation [11]. Therefore, exploring the m6A regulators may provide effective diagnostic markers and therapeutic targets for differentiation of RR-PTC. In this study, we investigate the most critical m6A regulator in differentiation of RR-PTC, and the potential molecular mechanisms are also elucidated.

## 2. Materials and Methods

### 2.1. Data Source

The RNA-Seq FPKM data target genes in thyroid cancer (*n* = 510) and non-cancer tissues (*n* = 58) were obtained from TCGA THCA datasets (https://portal.gdc.cancer.gov, accessed on 3 August 2020). The differential expression analysis was performed with the LIMMA package and the R function ‘wilcox.test’. The cutoff fold change was used as |logFC| > 0, and *p* < 0.05 was considered statistically significant. The correlation matrices of the target genes were constructed using the R package ‘corrplot’. A heatmap of the target genes was illustrated using the heatmap package. Gene set enrichment analysis (GSEA) analysis was performed using FPKM data from TCGA-THCA. The data was divided into a low-expression group and a high-expression group according to the median of IGF2BP2 expression in tumor samples. The pathway enrichment was performed using c2.cp.kegg.v7.4.symbols.gmt (http://www.GSEA-msigdb.org/GSEA/index.jsp accessed on 4 August 2020).

### 2.2. Tissue Specimen and Patient Information

Seventy lymph node or distant metastatic PTC (mPTC) patients from March 2017 to June 2020 were enrolled in this study, retrospectively. These patients did not receive any treatment before they underwent total thyroidectomy, but the metastatic lesions were not removed surgically before the initial course of ^131^I treatment. ^131^I treatment was performed during the six months after the thyroidectomy. Eighteen patients showed only lymph node metastases, fourteen patients showed only lung metastases, thirteen patients showed only bone metastases while twenty-five patients showed combined metastases or other sites (kidney or brain) metastases. The formalin-fixed paraffin-embedding specimens of the primary tumors of these patients, the post-therapeutic whole-body scan (Rx-WBS) as well as the clinical data were collected. The radioiodine uptake status was categorized as avid or ^131^I-nonavid according to the findings on Rx-WBS with SPECT/CT of metastatic lesions by visual analysis.

### 2.3. Cell Culture and Transfection

K1 with BRAF*^V^*^600*E*^ and TPC1 with RET/PTC rearrangement showed poor differentiated phenotypes, which can be utilized as RR-PTC models [12]. Cells were cultured in RPMI 1640 medium with 10% fetal bovine serum at 37 °C. Runt-related transcription factor 2 (RUNX2) siRNAs were transfected into cells using Lipofectamine 2000 (Invitrogen, Thermo Fisher Scientific, Waltham, MA, USA) according to the manufacturer’s instructions. For the stable silencing or overexpression IGF2BP2, lentivirus was constructed using pLKD-subcloned into the pcDNA3.1 vector (Invitrogen) according to the manufacturer’s instructions. An empty vector was used as negative control.

### 2.4. ^125^I Uptake Assay

Cells (1.5 × 10^5^) were seeded in six-well plates, and then shRNA or siRNAs were transfected into cells for 48 h. ^125^I uptake assay was performed by γ counter.

### 2.5. ^131^I Clonogenic Assay

Cells (5 × 10^2^) were seeded into six-well plates, and then shRNA or siRNAs were transfected into cells for 48 h. Regular culture medium with or without 20 μCi Na^131^I was used to culture cells for 6 h. The radioactive medium was replaced by regular culture medium and cultured about 2 weeks. Cells were fixed in methanol and stained by crystal violet. The number of colonies was counted.

### 2.6. Real-Time RT-qPCR Analysis

Cells (2.0 × 10^5^) were seeded in six-well plates and then IGF2BP2 shRNA or RUNX2 siRNAs were transfected into cells. RNA-Quick Purification Kit (Vazyme, Nanjing, China) was used for total RNA isolation from cells. 1 µg total RNA was converted to cDNA for RT-qPCR using HiScript II Q RT SuperMix for RT-qPCR (Vazyme, Nanjing, China). An Applied Biosystems 7500 Real-Time PCR System (Applied Biosystems) using AceQ qPCR SYBR Green Master Mix (Vazyme, Nanjing, China) was used for real-time quantitative RT-qPCR analysis. To standardize the input cDNA, β-actin was run in parallel as reference. The primer sequences are provided in Supplementary Table S1.

### 2.7. RNA Stability Assay

Cells (2.0 × 10^5^) were seeded in six-well plates, and then IGF2BP2 shRNA was transfected into cells for 48 h. Actinomycin D (Sigma-Aldrich, St. Louis, MO, USA) at 5 μg/mL was added into culture to inhibit gene transcription at 0 h, 3 h, and 6 h, and cells were collected for RT-qPCR.

### 2.8. Western Blot Assay

Cells (2.0 × 10^5^) were seeded in six-well plates and then IGF2BP2 shRNA or RUNX2 siRNAs were transfected into cells. After cells were lysed, the total protein was subjected to sodium dodecyl sulfate-polyacrylamide gel electrophoresis (EpiZyme, Shanghai, China) and then transferred to PVDF membranes (0.45 µmol; Millipore, Burlington, MA, USA). The membranes were blocked in 5% non-fat milk for 1h and immunoblotted with the primary antibodies for 24 h. Primary antibodies, including RUNX2, NIS (Abcam, Cambridge, MA, USA), and GAPDH (Cell Signaling Technology, Danvers, MA, USA). Membranes were then hybridized with species-specific HRP-conjugated antibodies (1:5000; Cell Signaling Technology) for 1 h. The membrane was subsequently washed 3 times in TBS/T. Finally, the ECL chromogenic agent was employed for exposure and imaging with a membrane scanner.

### 2.9. RNA Immunoprecipitation (RIP) Assay

RNA Immunoprecipitation Kit (Geneseed Biotech, Guangzhou, China) was used. Briefly, cells were collected and lysed by the RIP lysis buffer. Then the IGF2BP2 antibodies (5 µg) and a corresponding control, rabbit normal IgG, were added into the cleared lysates and incubated in the suspension with rotating magnetic beads overnight at 4 °C. Precipitate was digested with proteinase K buffer according to the manufacturer’s instructions. Co-immunoprecipitated RNA was isolated for RT-qPCR analysis.

### 2.10. Immunohistochemistry (IHC)

After 5-μm thyroid cancer sections treated with xylene, 100% alcohol, 95% alcohol, 85% alcohol, and 75% alcohol, successively, Tris/EDTA buffer was used for antigen retrieval. The sections were incubated with anti-IGF2BP2 (1:500 dilution in PBS) or anti-NIS (1:500 dilution in PBS) antibodies for 2 h. HRP-conjugated secondary (1:500 dilution in PBS) and 3,3′-diaminobenzidine were used to visualize the samples [13]. A semiquantitative method according to the staining intensity and the positive rate was used to score the staining. The intensity was defined as follows: 0, negative; 1, weak; 2, moderate; and 3, strong. The positive rate was defined as follows: 0, <1%; 1, 1–25%; 2, 26–50%; 3, 51–75%; and 4, 75–100%. IHC score = intensity × positive rate. IHC scores >6 and ≤6 were considered high and low expression, respectively [14].

### 2.11. Immunofluorescence Test

Cells were seeded in six-well chamber slides and transfected with siRNA for 48 h. 4% paraformaldehyde was used to fix the cells and 1% BSA was for blocking. Cells were then incubated with rabbit anti-NIS (1:100; Protein tech), and Goat Anti-Rabbit IgG H&L (Alexa Fluor^®^ 647) (1:100; Abcam), and DAPI.

### 2.12. Statistical Analysis

SPSS software (v. 24.0) was used for statistical analyses. Two-tailed Student’s *t* test was used to compare the difference between two groups. Expression correlation analysis was evaluated using the Pearson’s correlation coefficient. All graphs were generated by GraphPad Prism 6.0 software (GraphPad Software Inc., La Jolla, CA, USA). All *p* values were two-sided and *p* < 0.05 was considered statistically significant.

## 3. Results

### 3.1. Negative Association between Expression of IGF2BP2 and Differentiation Level of RR-PTC

**Figure 1:**
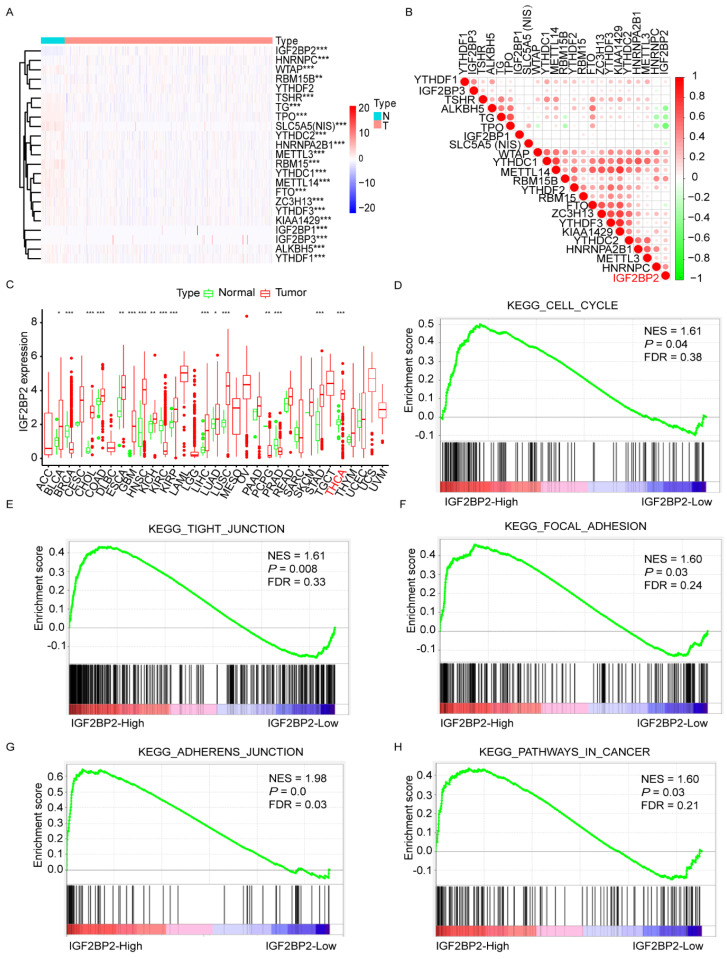
Negative association between the expression of IGF2BP2 and iodide-handling genes. (**A**) Expression of m6A RNA methylation regulators in thyroid cancer and normal thyroid. * *p* < 0.05, ** *p* < 0.01, *** *p* < 0.001. (**B**) Negative association between the expression of IGF2BP2 and iodide-handling genes. (**C**) Expression levels of IGF2BP2 in multiple cancers. GSEA plots showing that high IGF2BP2 expression is positively related to cell cycle (**D**), tight junction (**E**), focal adhesion (**F**), adherens junction (**G**), pathways in cancer (**H**).

Detailed analysis of public TCGA database from cBioPortal (http://cancergenome.nih.gov, accessed on 3 August 2020) showed that the expression of all the key m6A “writers”, “erasers”, and “readers”, and the iodide-handling genes (NIS, TPO, TSHR, and TG) were significantly different between normal thyroid tissues and thyroid cancer tissues (Figure 1A and Supplementary Table S2). Most of the key m6A regulators were positively correlated to iodide-handling genes in thyroid cancer, but IGF2BP2, HNRNPC, WTAP, and RBM15B were negatively correlated to iodide-handling genes, and the correlation between IGF2BP2 and iodide-handling gene was the strongest (Figure 1B and Supplementary Figure S1). Screening in multiple malignancies, IGF2BP2 was found to play oncogenic role in most of the malignancies including thyroid cancer (Figure 1C). Furthermore, GSEA with transcriptome datasets of primary thyroid cancer samples showed that high IGF2BP2 expression was positively correlated to genes related to the cell cycle, tight junction, focal adhesion, and adherens junction pathways in cancer (Figure 1D–H).

**Figure 2:**
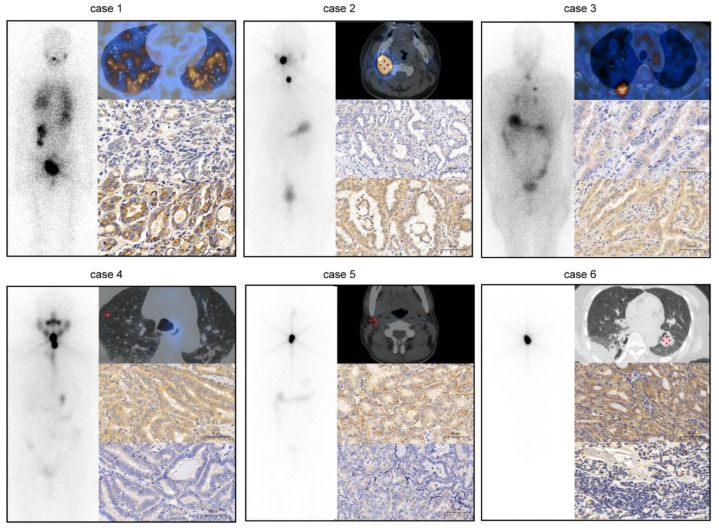
Post-therapeutic whole-body scan (Rx-WBS) with SPECT/CT after the initial course of ^131^I and immunohistochemistry of IGF2BP2 and NIS of primary tumor in Representative cases. Patients (case 1: follicular variant, case 2: classic variant, case 3: tall cell variant) with low expression of IGF2BP2 in primary tumors were likely to show ^131^I-avid lesions on Rx-WBS. Patients (case 4: tall cell variant, case 5: follicular variant, case 6: follicular variant) with high expression of IGF2BP2 in primary tumors were likely to show ^131^I-nonavid lesions on Rx-WBS. Scale bar: 50 µm.

According to IGF2BP2 level, clinicopathological data deriving from a total of 70 patients with mPTC were analyzed. IGF2BP2 was correlated with tumor size (*p* = 0.023), and status of ^131^I uptake (*p* = 0.015) (Table 1). Considering that NIS exerted critical role in restoring of ^131^I in PTC, the relationship between IGF2BP2 and NIS was further explored. Negative association between IGF2BP2 and NIS was noted in mPTCs. Compared to mPTCs with low IGF2BP2 level, those with high IGF2BP2 level were likely to be ^131^I-nonavid. Representative cases were shown in Figure 2. These results indicated that IGF2BP2 might be associated with differentiation level of PTC.

### 3.2. Knockdown of IGF2BP2 Enhances Differentiation of RR-PTC Cells

**Figure 3:**
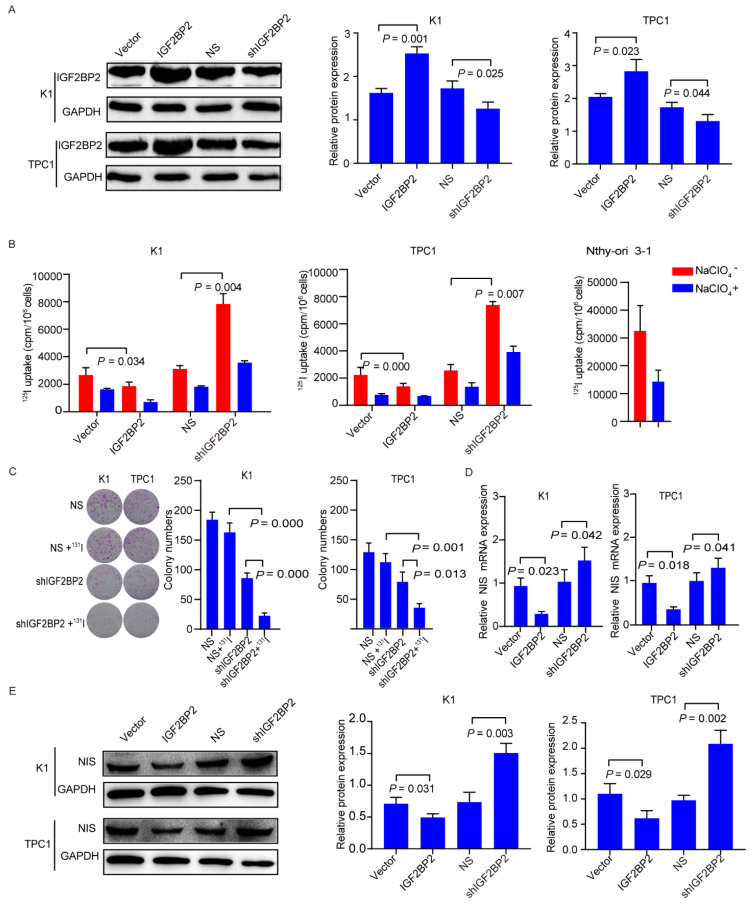
Targeting IGF2BP2 promotes differentiation of PTC. (**A**) Successful transfection in K1 and TPC1 cells with shIGF2BP2 and IGF2BP2 overexpression demonstrated by Western blot. (**B**) ^125^I uptake in K1 and TPC1 cells with or without transfection with IGF2BP2 overexpression or knockdown. (**C**) ^131^I clonogenic assay in K1 and TPC1 cells with or without transfection with shIGF2BP2. (**D**) NIS mRNA expression in K1 and TPC1 cells with or without transfection with IGF2BP2 overexpression or knockdown. (**E**) NIS protein expression in K1 and TPC1 cells with or without transfection with IGF2BP2 overexpression or knockdown.

In order to investigate the biological role of IGF2BP2 in dedifferentiation of PTC, successful transfection with lentivirus on RR-PTC cells (K1 and TPC1) was obtained, which showed robust decrease or increase in the expression of IGF2BP2 on Western blots (Figure 3A, the uncropped Western blot figures could be found in the Supplementary Figure S2A). Results elucidated that overexpression of IGF2BP2 induced ^125^I uptake decrease and suppression of IGF2BP2 induced ^125^I uptake increase (all *p* < 0.05) (Figure 3B). Moreover, compared to NS group, knockdown of IGF2BP2 alone resulted in a significant drop in the numbers of colonies, which decreased significantly further after ^131^I treatment (all *p* < 0.05) (Figure 3C). In addition, NIS expression was also evaluated through targeting IGF2BP2. NIS mRNA expression was significantly decreased after overexpression of IGF2BP2. Whereas, NIS mRNA expression was slightly enhanced after knockdown of IGF2BP2, which is in line with the findings of bioinformatic analyses (Figure 3D). In protein levels, NIS protein expression was decreased after overexpression of IGF2BP2and elevated significantly after knockdown of IGF2BP2 (Figure 3E, the uncropped Western blot figures can be found in the Supplementary Figure S2B). The above data suggested that depletion of IGF2BP2 alone is dispensable for the differentiation of RR-PTC.

### 3.3. IGF2BP2 Stabilizes RUNX2 mRNA via Binding to 3′-Untranslated Region (UTR)

**Figure 4:**
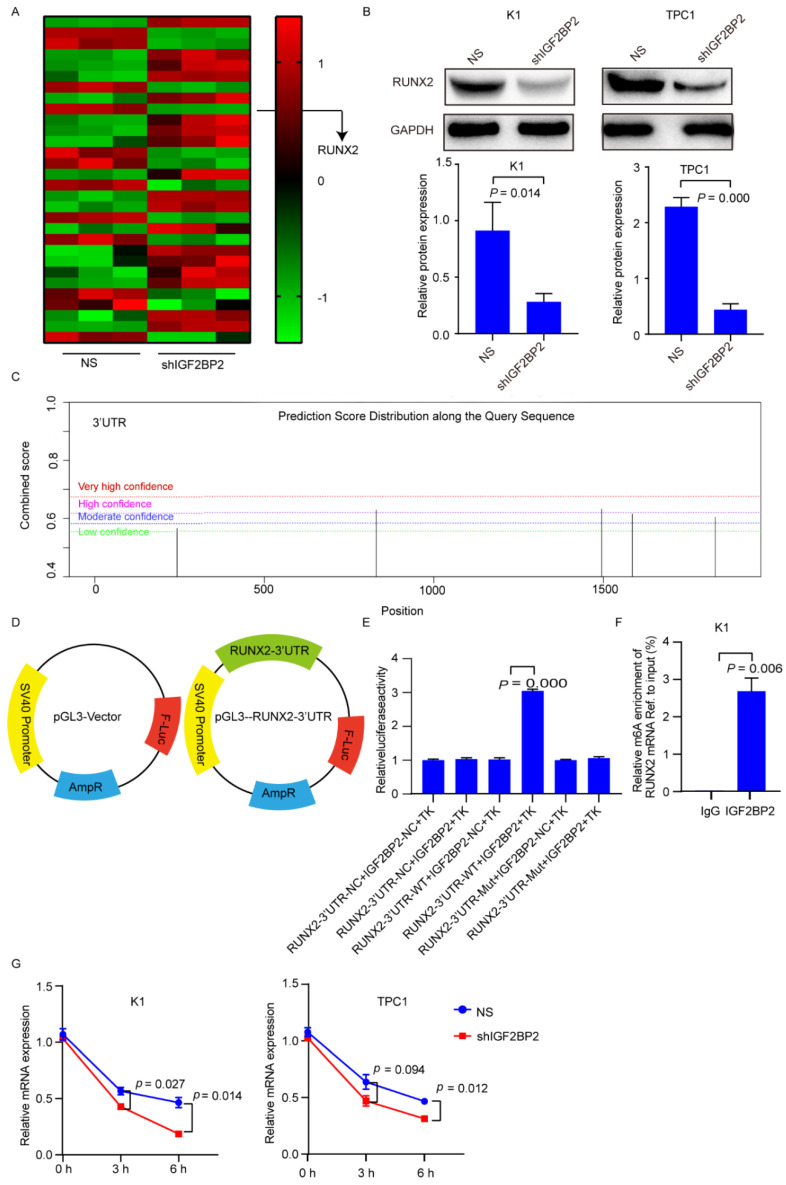
RUNX2 is a potential target of IGF2BP2. (**A**) Heatmap of differentially expressed genes, of which RUNX2 is associated with cell differentiation in K1 cells via knockdown of IGF2BP2. (**B**) RUNX2 protein expression in K1 and TPC1 cells with or without knockdown of IGF2BP2. (**C**) Prediction of potential m6A binding sites in 3′-UTR of RUNX2 mRNA. (**D**) Graphical explanation for construction of luciferase reporters with wild-type or mutant (m6A motif mutated) sequence of RUNX2-3′-UTR inserting into a pGL3 vector. (**E**) Relative luciferase activities in TPC1 cells with stably expressing IGF2BP2 or vector co-transfected with RUNX2-3′-UTR wild-type or mutant luciferase reporters. (**F**) Relative enrichment of RUNX2 mRNA associated with IGF2BP2 protein using anti-IgG and anti-IGF2BP2 antibodies. (**G**) mRNA stability of RUNX2 in K1 and TPC1 cells with or without knockdown of IGF2BP2.

To look into the potential targets for IGF2BP2, we underwent RNA-seq in IGF2BP2 knockdown K1 cells. We found 5418 differentially expressed genes (DEGs) (*p* value < 0.01). A total of 607 DEGs were associated with cell differentiation, in which 39 were enriched in thyroid cancer regulation. The top genes were shown in Figure 4A. The correlation between the top 30 DEGs and NIS based on TCGA database are shown Supplementary Table S3. In these DEGs, RUNX2 was positively correlated with IGF2BP2 and negatively associated with NIS in thyroid cancer. Therefore, we selected RUNX2 as the target gene for IGF2BP2 to influence the expression of NIS.

To investigate the relationship between IGF2BP2 and RUNX2, RUNX2 mRNA and protein levels were evaluated in K1 and TPC1 cells with IGF2BP2 suppression or overexpression by lentivirus transfection. RUNX2 protein expression was decreased in K1 and TPC1 cells with IGF2BP2 suppression (Figure 4B, the uncropped Western blot figures can be found in the Supplementary Figure S2C). m6A sites based on the presence of the DRACH (D = A, G or U; R = A or G; H = A, C or U) consensus motifs in the coding sequence (CDS) and 3′-UTR sequence of RUNX2 were predicted by SRAMP (http://www.cuilab.cn/sramp/, accessed on 13 June 2021). Five predicted m6A sites with low or moderate confidence were distributed in CDS (Supplementary Figure S3A). Four predicted m6A sites were distributed in 3′-UTR, of which two predicted m6A sites were with high or very high confidence (Figure 4C). Next, the pGL3 dual-luciferase reporter fused with the CDS and 3′-UTR fragments of RUNX2 were established, respectively (Supplementary Figure S3B and Figure 4D). Insertion of CDS of the RUNX2 gene demonstrated no difference of luciferase activity in control and IGF2BP2 stably expressing cells (Supplementary Figure S3C). On the contrary, the luciferase activity of the construct containing 3′-UTR was significantly enhanced in TPC1 cells with IGF2BP2 stably expression (Figure 4E). These data indicate that IGF2BP2 regulates RUNX2 via its 3′-UTR. Furthermore, the direct interaction between IGF2BP2 and RUNX2 mRNA was shown by RIP-qPCR (Figure 4F). Inhibiting IGF2BP2 shortened RUNX2 half-life (Figure 4G). Overall, these findings illustrate that IGF2BP2 maintains RUNX2 stability by binding to m6A methylation of 3′-UTR of RUNX2.

### 3.4. Inhibiting RUNX2 Promotes Differentiation of RR-PTC

**Figure 5:**
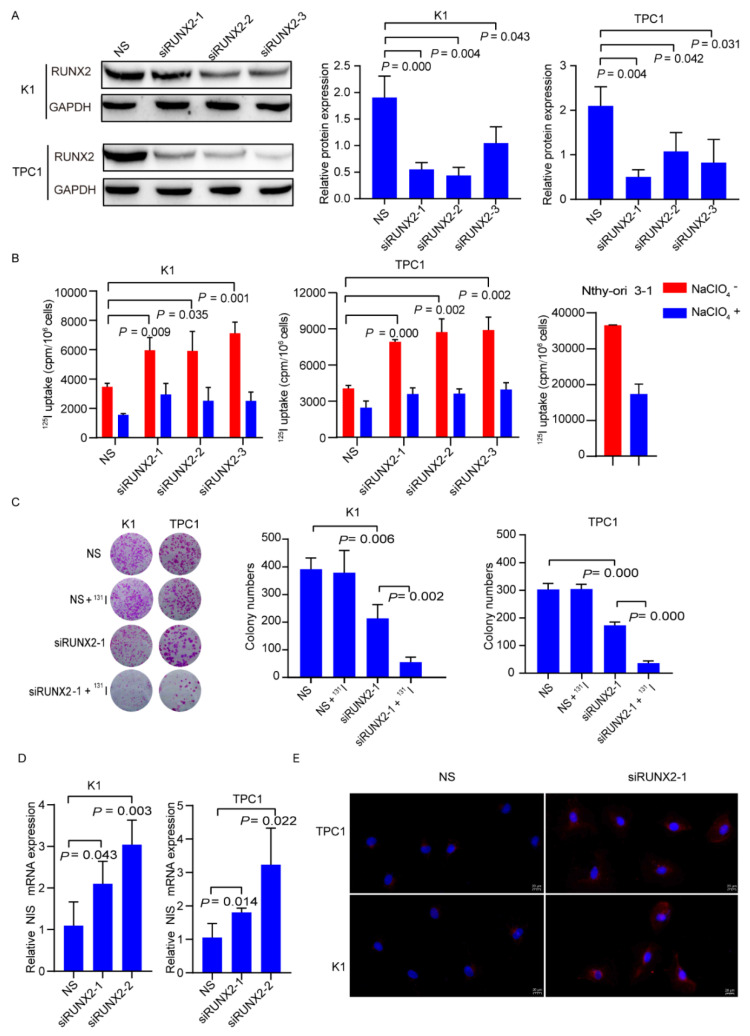
Targeting RUNX2 enhances differentiation of RR-PTC cells. (**A**) Successful transfection with siRUNX2-1, siRUNX2-2, and siRUNX2-3 demonstrated by Western blot. (**B**) ^125^I uptake in K1 and TPC1 cells with or without transfection with siRUNX2-1, siRUNX2-2, and siRUNX2-3. (**C**) ^131^I clonogenic assay in K1 and TPC1 cells with or without transfection with or without siRUNX2-1. (**D**) NIS mRNA expression in K1 and TPC1 cells with or without transfection with siRUNX2-1 and siRUNX2-2. (**E**) NIS membrane localization in K1 and TPC1 cells with or without transfection with siRUNX2-1. Scale bar: 20 µm.

To evaluate the role of RUNX2 in differentiation of RR-PTC cells, successful transfection with siRNAs (siRUNX2-1, siRUNX2-2, siRUNX2-3) was obtained (Figure 5A, the uncropped Western blot figures could be found in the Supplementary Figure S2D). Suppression of RUNX2 induced ^125^I uptake increase, in detail, 1.71-, 1.70-, and 2.05-fold higher in K1 cells (all *p* < 0.05); 1.96-, 2.15-, and 2.20-fold higher in TPC-1 cells (all *p* < 0.05) (Figure 5B). Moreover, compared with the NS group, representative siRNA (siRUNX2-1) transfection alone resulted in a significant drop in the number of colonies, which further decreased significantly after ^131^I treatment in PTC cells (all *p* < 0.05) (Figure 5C). Similarly, NIS mRNA expression levels were distinctly enhanced after the knockdown of RUNX2 by transfection with siRUNX2-1 and siRUNX2-2(Figure 5D). NIS protein membrane localization was enhanced with knockdown of RUNX2 (Figure 5E).

Given that targeting IGF2BP2 promotes differentiation of RR-PTC via destabilizing RUNX2 mRNA, we proposed that co-targeting IGF2BP2 and RUNX2 could obtain robust differentiation of RR-PTC. As we expected, compared to the cells with knockdown of IGF2BP2 alone, ^125^I uptake (Supplementary Figure S4A) was significantly enhanced, the number of colonies (Supplementary Figure S4B) obviously declined, and NIS protein expression (Supplementary Figure S4C, the uncropped Western blot figures can be found in the Supplementary Figure S2E) was enhanced with co-suppressed IGF2BP2 and RUNX2. Taken together, these findings confirmed that inhibiting RUNX2 promotes differentiation of RR-PTC.

### 3.5. RUNX2 Downregulates NIS Expression via Binding to Promoter of NIS

**Figure 6:**
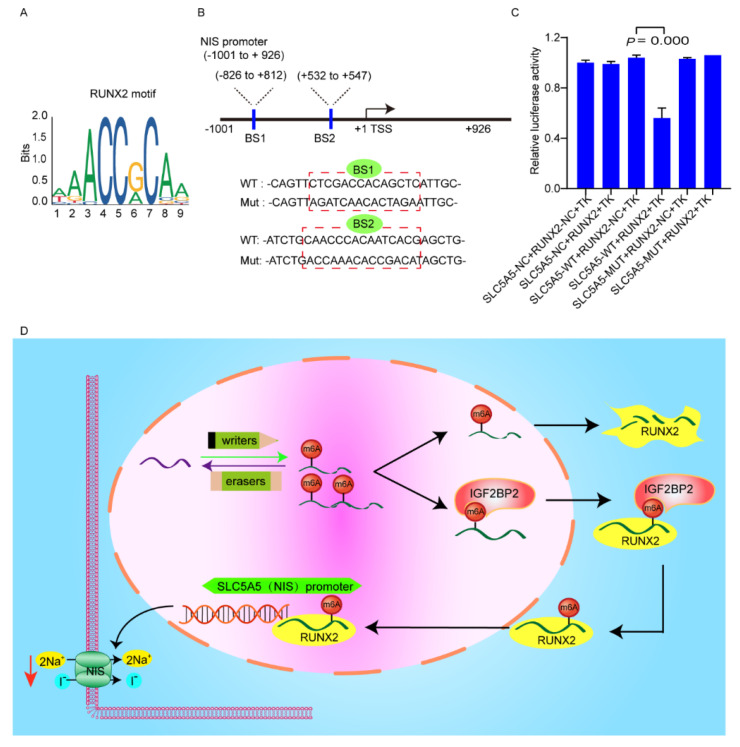
RUNX2 binds to the promoter region of NIS. (**A**) The consensus motif of RUNX2 is illustrated based on the JASPER database. (**B**) Graphical explanation for construction of luciferase reporters with wild-type or mutant sequence of promoter region of NIS inserting into a pGL3 vector. (**C**) Relative luciferase activities in TPC1 cells with stably expressing RUNX2 or vector co-transfected with the NIS-luciferase reporters. (**D**) Working model of IGF2BP2/RUNX2-mediated dedifferentiation of PTC.

RUNX2 may bind to the promoter region of NIS mRNA based on JASPAR database (http://jaspardev.genereg.net/, accessed on 6 July 2021) analysis (Figure 6A). To determine whether RUNX2 binds to the NIS promoter region, pGL3 dual-luciferase reporter fused with the promoter region of NIS was established (Figure 6B). The luciferase activity of the construct containing 3′-UTR was significantly reduced in cells stably expressing RUNX2 compared to the vector cells (Figure 6C). A working model of IGF2BP2-mediated dedifferentiation of PTC is shown in Figure 6D. The above findings demonstrated that RUNX2 downregulates NIS expression via binding to promoter of NIS.

## 4. Discussion

To date, accumulating evidence indicates that differentiation/redifferentiation therapy could play a significant role in RR-PTC management. In this study, interrogation of TCGA thyroid cancer datasets revealed that the expression of IGF2BP2 was negatively associated with that of thyroid-specific iodine-handling genes. RNA-seq and IGF2BP2-RIP-qPCR assays identified RUNX2 as a direct target of IGF2BP2. We showed that IGF2BP2 positively regulated RUNX2 mRNA stability in an m6A-dependent manner. Functional studies validated that RUNX2 was a pivotal contributor to the dedifferentiation effect of IGF2BP2, as RUNX2 binds to the promoter region of NIS, downregulating its expression.

IGF2BP2 has been identified as a regulator of differentiation, and serves posttranscriptional regulation to participate in the differentiation of cancer and cancer stem cells [15], such as hepatocellular carcinoma [16], liposarcoma [17], glioblastoma [18], etc. Previous studies have demonstrated that IGF2BP2 was upregulated in thyroid cancer and strongly correlated with disease-free survival and clinical phenotypes of PTC [19,20]. Although the function of IGF2BP2 in thyroid cancer was totally based on the public database analysis and did not reveal the underlying mechanism of the role of IGF2BP2, they do indicate that IGF2BP2 is a gene worthy of further analysis to account for the pathophysiological process of thyroid cancer. Based on GESA analyses, high IGF2BP2 was positively correlated with several biological processes such as cell cycle, tight junction and focal adhesion, which were confirmed as being associated with cell differentiation in previous studies [21,22,23]. Inspiringly, we found that mPTC patients with high expression of IGF2BP2 benefitted less from ^131^I treatment, indicating that such disease was apt to progress to RR-PTC, which was in line with findings of online database analyses. We further showed that suppression of IGF2BP2 improves ^125^I uptake, and ^131^I toxicity. IGF2BP2 knockdown slightly enhanced NIS mRNA level, but significantly increased NIS protein level, indicating that a low level of NIS transcription induced by IGF2PB2 knockdown resulted in a robust protein translation. Therefore, understanding the functions of IGF2BP2 may facilitate the development of new differentiation strategy of RR-PTC.

IGF2BP2 possessed six RNA-binding domains (RBDs), including two N-terminal RRMs and four C-terminal KH domains [24]. RBDs determine the functional diversity of IGF2BP2 and mediate the recruitment of other RNA binding proteins to the protein-RNA complexes due to their flexible sequence and structure [15,25]. IGF2BP2 as an m6A reader, binds to the m6A motifs of targets and regulates RNA location, stability and translation. To date, a number of client mRNAs for IGF2BP2 have been identified in several cancers, including insulin-like growth factor 1 receptor in prostate cancer [26], Zinc-finger E-box binding protein 1 in gastric cancer [27], High-mobility group A1 in colorectal cancer [28], etc. The m6A motifs are typically enriched in the 3′-UTR and CDS, regulating precursor mRNA maturation, translation, and degradation [29]. In this study, IGF2BP2 integrated with the 3′-UTR of RUNX2, consequently destabilizing the stability of RUNX2 mRNA.

As a member of RUNX family, RUNX2 is a critical regulator and master organizer in bone development and several malignances, including lung cancer, breast cancer, pancreatic cancer, liver, prostate cancer, ovarian cancer, etc. [30,31]. In thyroid cancer, RUNX2 acts as an oncogene [32], mediating aggressive features of tumor and controlling migration and invasiveness [33]. In this study, silencing RUNX2 resulted in differentiation of PTC, and synergized the differentiation effect of targeting IGF2BP2. Mechanistically, RUNX2 as a transcription factor, can bind to the promoter of target genes to regulate their activities. In this study, RUNX2 inhibited NIS expression via binding to the promoter of NIS. Taken together, the exploring of RUNX2 provides new targets for the differentiation of PTC.

## 5. Conclusions

Our study demonstrated that IGF2BP2 has the potential to become a novel biomarker of dedifferentiation and could serve as a therapeutic target for differentiation of RR-PTC.

## Figures and Tables

**Table 1:** Clinicopathological features of patients with metastatic papillary thyroid cancer according to the expression of IGF2BP2 (*n* = 70).

Characteristic	High (%)	Low (%)	*p*-Value
Age			0.689
≥55 years	22 (56.4%)	16 (51.6%)	
<55 years	17 (43.6%)	15 (48.4%)	
Sex			0.313
Female	26 (66.7%)	17 (54.8%)	
Male	13 (33.3%)	14 (45.2%)	
Size			0.023 *
≤2.0 cm	8 (20.5%)	11 (35.5%)	
2.0~4.0 cm	11 (28.2%)	14 (45.2%)	
>4.0 cm	20 (51.3%)	6 (19.3%)	
Metastatic site(s)			0.233
Lymph node-only	8 (20.5%)	10 (32.3%)	
Lung-only	11 (28.2%)	3 (9.6%)	
Bone-only	6 (15.4%)	7 (22.6%)	
Others or combined	14 (35.9%)	11 (35.5%)	
Rx-WBS			0.015 *
^131^I-avid	14 (35.9%)	21 (67.7%)	
^131^I-nonavid	25 (64.1%)	10 (32.3%)	
NIS expression			0.032 *
Low	23 (59.0%)	10 (32.3%)	
High	16 (41.0%)	21 (67.7%)	

^131^I, radioiodine; Rx-WBS, post-therapeutic whole-body scan; NIS, sodium iodine transporter; * Statistical significant difference.

## Data Availability

The data presented in this study are available on request from the corresponding author.

## Supplementary Materials

The following supporting information can be downloaded at: https://www.mdpi.com/article/10.3390/cancers14051268/s1, Figure S1: Negative association between the expression of IGF2BP2 and iodide-handling genes with correlation coefficient; Figure S2: The uncropped western blot figures; IGF2BP2 does not bind to the CDS of Runx2; Figure S4: Targeting IGF2BP2 and RUNX2 have robust cell differentiation effect in PTC; Table S1: The primer sequences; Table S2: Expression of m6A key regulators and iodide-handling genes in normal thyroid tissues and thyroid cancer based on TCGA database; Table S3: Pearson correlation between SLC5A5 and top 30 genes from RNA-seq associated with cell differentiation of malignant cancers.
