# Supplementary material for: Targeting IGF2BP2 Promotes Differentiation of Radioiodine Refractory Papillary Thyroid Cancer via Destabilizing RUNX2 mRNA
Source: Cancers (Basel). 2022 Mar 1;14(5):1268. doi: 10.3390/cancers14051268 (PMC8909796; doi:10.3390/cancers14051268)
Supplement: Supplementary file 1 [file cancers-14-01268-s001.zip › cancers-1548479-supplementary.pdf]

cancers MDPI

# Targeting IGF2BP2 Promotes Differentiation of Radioiodine Refractory Papillary Thyroid Cancer via Destabilizing RUNX2 mRNA

**Ri Sa, Rui Liang, Xian Qiu, Ziyan He, Zhiyan Liu and Libo Chen**

**Table S1.** The primer sequences.

| gene | sequences |
|---|---|
| SLC5A5-S | CTGCCCCAGACCAGTACATGCC |
| SLC5A5-A | TGACGGTGAAGGAGCCCTGAAG |
| IGF2BP2-S | AACCCCAAAGAAGAAGTGAAGC |
| IGF2BP2-A | TTTGGTCACGAGGCACGAT |
| β-actin-S | TCTACAATGAGCTGCGTGTG |
| β-actin-A | TAGATGGGCACAGTGTGGGT |

**Table S2.** Expression of m6A key regulators and iodide-handling genes in normal thyroid tissues and thyroid cancer based on TCGA database.

| gene | normal thyroid tissues Mean | thyroid cancer Mean | logFC | *p* Value |
|---|---|---|---|---|
| YTHDC1 | 16.475244 | 12.36228568 | -0.414354322 | 4.86E-19 |
| TSHR | 145.5524059 | 100.3074123 | -0.537110468 | 3.21E-14 |
| TG | 7096.331946 | 3053.501668 | -1.216608863 | 2.48E-21 |
| FTO | 5.22394037 | 3.930772179 | -0.410325679 | 6.69E-16 |
| ALKBH5 | 38.92788526 | 34.64486202 | -0.168162563 | 4.24E-08 |
| METTL14 | 5.241399729 | 3.951812242 | -0.407437735 | 1.92E-19 |
| SLC5A5 | 20.99236121 | 3.699198362 | -2.50457988 | 1.04E-26 |
| HNRNPC | 44.56087334 | 48.77368184 | 0.130325375 | 5.79E-05 |
| TPO | 666.2346093 | 185.7948459 | -1.84231982 | 4.61E-23 |
| YTHDF1 | 22.62329119 | 20.68445037 | -0.129262202 | 2.20E-06 |
| RBM15B | 11.01082181 | 11.68894403 | 0.086222454 | 0.003823026 |
| YTHDF3 | 12.07129406 | 10.18577346 | -0.245024808 | 3.18E-08 |
| YTHDC2 | 3.200695013 | 2.698269876 | -0.246350561 | 1.78E-06 |
| YTHDF2 | 16.99331941 | 16.93274625 | -0.005151714 | 0.904226929 |
| HNRNPA2B1 | 93.17716572 | 79.5028424 | -0.228970006 | 1.03E-09 |
| IGF2BP1 | 0.014391499 | 0.033158128 | 1.20414574 | 2.35E-07 |
| RBM15 | 2.681661904 | 1.841058101 | -0.542592201 | 8.64E-24 |
| KIAA1429 | 5.489612952 | 4.709760701 | -0.221050675 | 3.87E-08 |
| IGF2BP3 | 0.139347652 | 0.252216971 | 0.855976661 | 1.57E-10 |
| METTL3 | 7.094790945 | 6.137485522 | -0.209112458 | 8.14E-05 |
| WTAP | 17.36785137 | 14.499494 | -0.26041673 | 1.74E-09 |
| IGF2BP2 | 3.42588301 | 12.25571513 | 1.838906878 | 1.78E-24 |
| ZC3H13 | 10.76703674 | 8.151836341 | -0.401424259 | 2.10E-13 |

**Table S3.** Pearson correlation between SLC5A5 and top 30 genes from RNA-seq associated with cell differentiation of malignant cancers.

| | R | *p*Value |
|---|---|---|
| CCND1 | -0.086 | 0.051 |
| THRB | -0.017 | 0.7 |
| NOTCH3 | 0.013 | 0.77 |
| PLCG2 | 0.065 | 0.14 |
| MMP14 | -0.027 | 0.55 |

| | | |
|---|---|---|
| GPX1 | -0.052 | 0.24 |
| HLA-B | -0.054 | 0.22 |
| SLC9A1 | 0.25 | 7.60E-09 |
| RUNX2 | -0.099 | 0.041 |
| EGR1 | -0.022 | 0.62 |
| GNA12 | -0.0028 | 0.95 |
| PPARG | 0.0086 | 0.85 |
| MAPK3 | 0.17 | 0.00015 |
| ITGB3 | 0.092 | 0.037 |
| TCF7 | -0.047 | 0.29 |
| SIN3A | -0.009 | 0.84 |
| FOXO1 | -0.023 | 0.61 |
| MEF2C | 0.051 | 0.25 |
| THRA | 0.071 | 0.11 |
| PIK3R1 | -0.027 | 0.54 |
| HDAC1 | -0.011 | 0.8 |
| MAPK1 | -0.029 | 0.51 |
| NOTCH1 | 0.11 | 0.01 |
| SRC | -0.011 | 0.014 |
| RCAN1 | 0.0019 | 0.97 |
| GNA13 | -0.0011 | 0.98 |
| TCF7L2 | -0.036 | 0.41 |
| KAT2A | -0.073 | 0.099 |
| GATA3 | -0.027 | 0.55 |
| RXRA | 0.14 | 0.0013 |

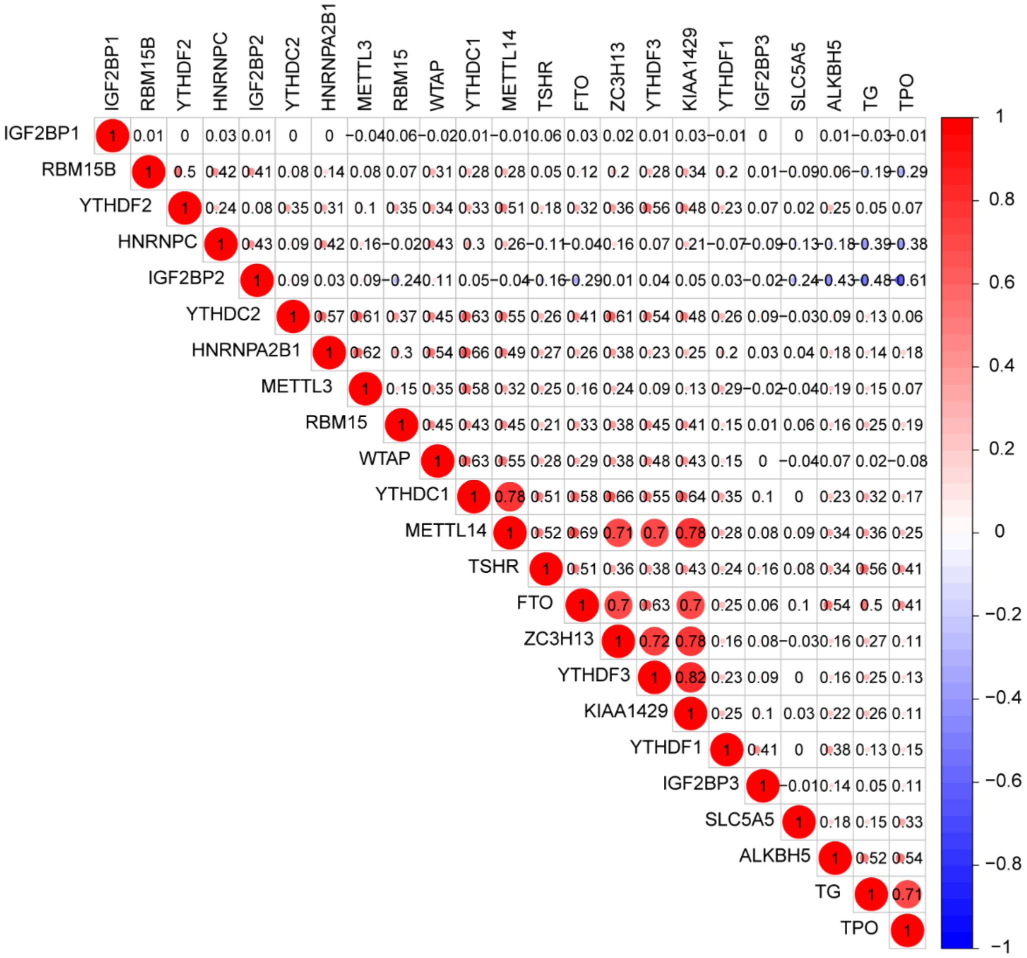


**Figure S1.** Negative association between the expression of IGF2BP2 and iodide-handling genes with correlation coefficient.

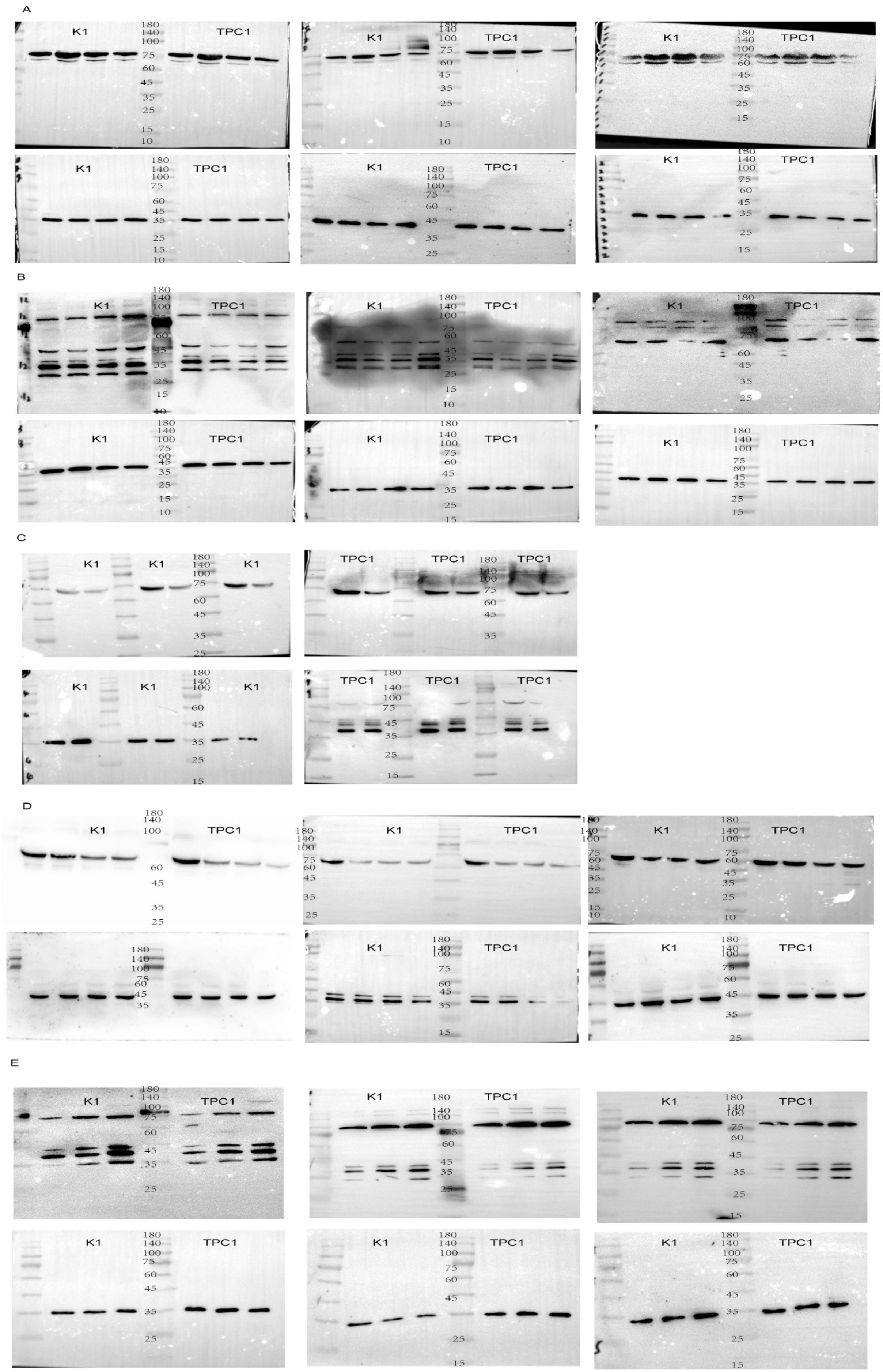
A
K1
TPC1
B
K1
TPC1
C
K1
TPC1
D
K1
TPC1
E
K1
TPC1

**Figure S2.** The uncropped Western blot figures for Figure 3A (A), Figure 3E (B), Figure 4B (C), Figure 5A (D), supplementary Figure 4C (E).

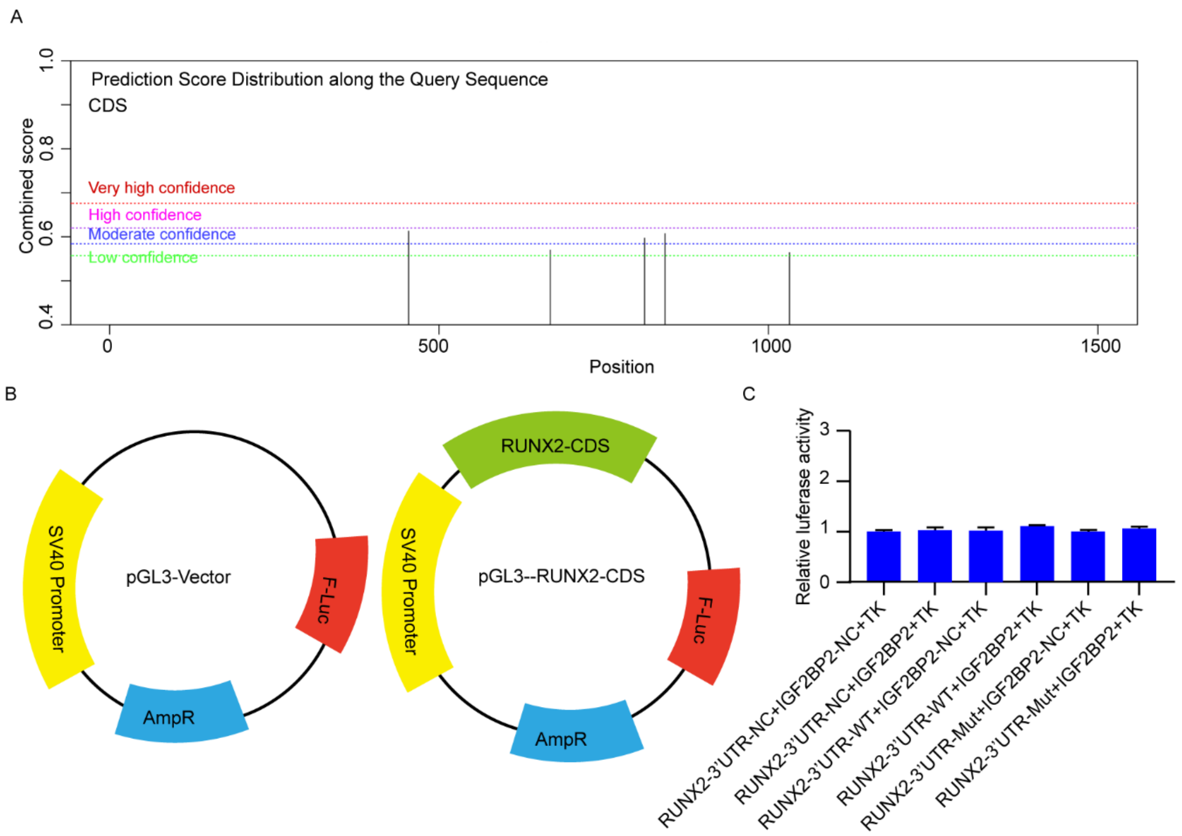


**Figure S3.** IGF2BP2 does not bind to the CDS of RUNX2. (A) Prediction of potential m6A binding sites in CDS of RUNX2 mRNA. (B) Graphical explanation for construction of luciferase reporters with wild-type or mutant (m6A motif mutated) sequence of RUNX2–CDS inserting into a pGL3 vector. (C) Relative luciferase activities in TPC1 cells with stably expressing IGF2BP2 or vector co-transfected with RUNX2–CDS wild-type or mutant luciferase reporters.

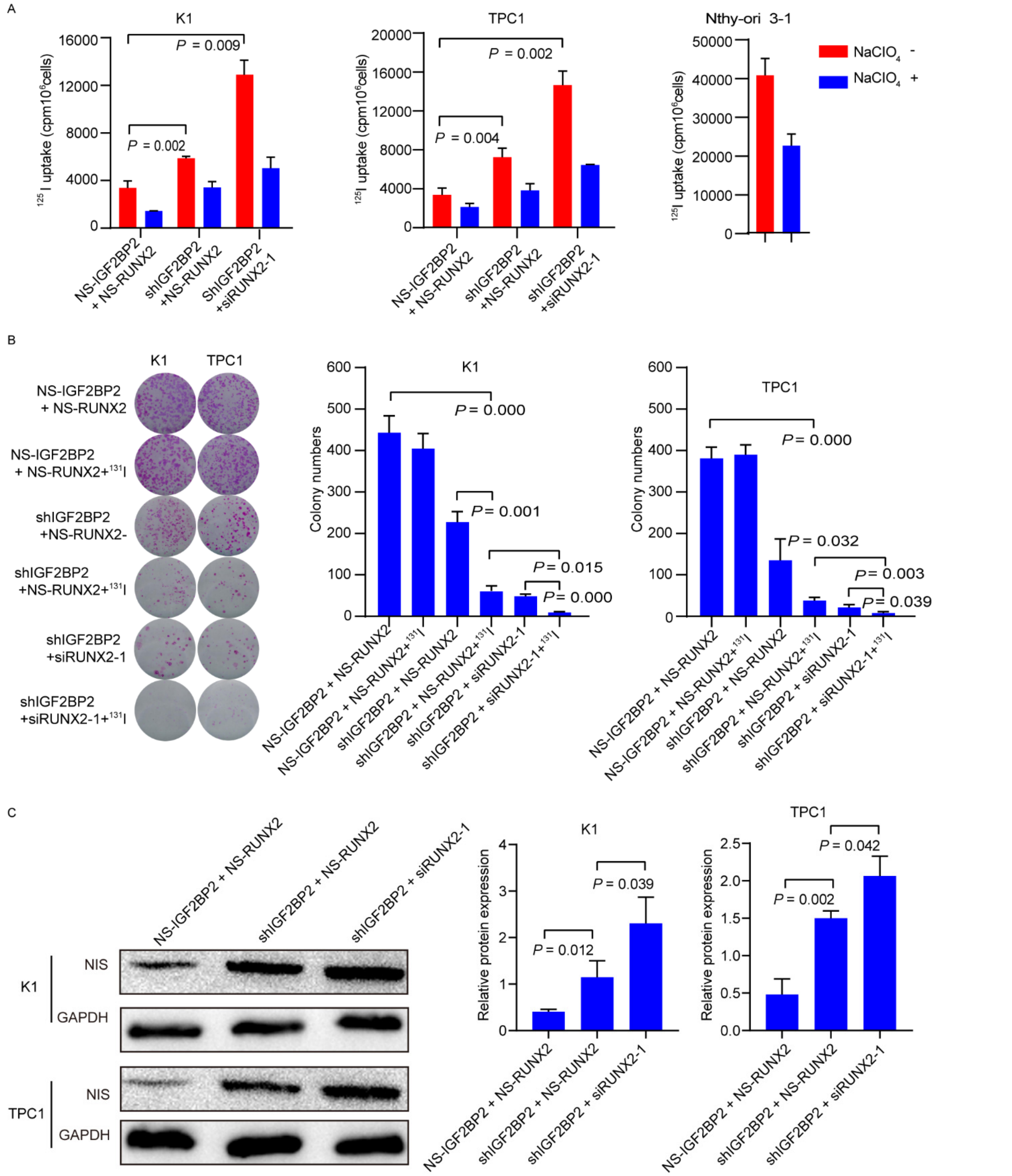


**Figure S4.** Targeting IGF2BP2 and RUNX2 have robust cell differentiation effect in PTC. (A) $^{125}I$ uptake in K1 and TPC1 cells with or without co-transfection with shIGF2BP2 and siRUNX2-1. (B) $^{131}I$ clonogenic assay in K1 and TPC1 cells with or without co-transfection with shIGF2BP2 and siRUNX2-1. (C) NIS protein expression in K1 and TPC1 cells with or without co-transfection with shIGF2BP2 and siRUNX2-1.
